# The Interfacial Structure and Adhesion Mechanism of *N*-(2-Aminoethyl)-3-aminopropyltrimethoxysilane and Epoxy Modified Silicone Tie-Coating to Epoxy Primer

**DOI:** 10.3390/polym13173001

**Published:** 2021-09-04

**Authors:** Hongyang Zhang, Zhanping Zhang, Yuhong Qi, Qiang Yang

**Affiliations:** Department of Materials Science and Engineering, Dalian Maritime University, Dalian 116026, China; zhy686@dlmu.edu.cn (H.Z.); yuhong_qi@dlmu.edu.cn (Y.Q.); 1120180341yq@dlmu.edu.cn (Q.Y.)

**Keywords:** silicone tie-coating, epoxy, *N*-(2-Aminoethyl)-3-aminopropyltrimethoxysilane, polydimethylsiloxane, adhesion

## Abstract

The matching application of silicone antifouling coating and epoxy primer is a major problem in engineering. Novel epoxy-modified silicone tie-coating was prepared to tie epoxy primer and silicone antifouling coating. Firstly, *N*-(2-Aminoethyl)-3-aminopropyltrimethoxysilane was mechanically mixed with bisphenol A epoxy resin to form silylated epoxy resin, then the silylated epoxy resin was uniformly mixed with hydroxy-terminated polydimethylsiloxane and a curing agent and catalyst for coating. An infrared spectrometer, differential scanning calorimeter and tensile tests were used to investigate the chemical structure, phase transition temperature and mechanical properties of the tie-coatings. The interlaminar adhesion of the matching coating system was tested and analyzed by a peel-off test and a shear test. Fracture morphology was observed by scanning using an electron microscope. The results showed that crosslinking density of the tie-coating, the elastic modulus and the tensile strength of the coating increased with an increasing epoxy content, but fracture elongation decreased. The shear strength of the matching coating system is 0.37 MPa, and it shows a good tie performance. The maximum anti-peeling rate of the tie-coating on the epoxy primer reaches 100%.

## 1. Introduction

For a long time, marine ships have been facing the harm of marine corrosion and marine fouling. Given this problem, the use of functional coatings has become an effective solution. However, there is no reliable coating that can provide excellent anti-corrosion and anti-fouling properties at the same time. In most cases, the matching application of anti-corrosion coating and anti-fouling coating is the conventional coating method of a modern ship surface. Generally, the ship coating process is first to apply anti-corrosion coating on the surface of the substrate, and then anti-fouling coating on the surface of the anti-corrosion coating, to achieve the purpose of anti-corrosion and anti-fouling. An epoxy primer is a common anti-corrosion coating, which has good corrosion resistance, good mechanical strength and good adhesion with the substrate. It is an important coating in ship protection [1,2,3]. There are many kinds of antifouling coatings [4]. The mature antifouling coatings include low surface energy antifouling coatings [5], self-polishing antifouling coatings [6] and amphiphilic antifouling coatings [7,8], etc. These coatings are widely used, and their functional effects and economic benefits are indispensable. However, with the enhancement of people’s awareness of environmental protection, the environmental attributes of coatings have become a necessary consideration when people choose them. Some coatings that are harmful to the environment have been gradually eliminated, and environmentally friendly coatings have become popular coatings in the market. Hydroxy-terminated polydimethylsiloxane (PDMS) is a common silicone polymer [9] and is commonly used to fabricate superhydrophobic surfaces because of its low surface energy. In addition to being inexpensive, PDMS also shows a good thermal stability, oxidative stability and non-toxicity [10,11]. Silicone antifouling coatings prepared from PDMS are widely used due to their advantages of non-toxic, economical and excellent antifouling effect [12]. However, its adhesion on the epoxy primer is poor, and it easily falls from the epoxy primer, resulting in a failure of the antifouling effect [13,14].

In response to this problem, a more mature solution is to connect the epoxy primer with a silicone antifouling coating by using tie-coating. Tie-coating can be divided into two types according to the connection mechanism. One type is silane coupling agent-type tie-coatings. The silane coupling agent is introduced into the silicone coating and the coating can be given the function of connecting with the epoxy primer. In our previous study [13], we introduced a certain amount of *N*-(2-Aminoethyl)-3-aminopropyltrimethoxysilane (DAMO) into PDMS by mechanical mixing, and the coating was cured under the action of the catalyst and a curing agent. The coating showed a good tie performance. The other type is a silicone-epoxy polymer-type tie-coatings. This coating improves adhesion through the epoxy component in the polymer. Compared with silane coupling coatings, it has advantages in mechanical properties and improves the protective effect on the substrate to a certain extent. In the initial stage, researchers physically blended epoxy resin and silicone resin; however, due to the huge difference in solubility between the two resins [14,15], the two resins appeared to have phase separation [16], which affected the performance of the coating. Many efforts have been made to improve the compatibility between the epoxy resin and silicone resin; for example, adding solubilizer [17], grafting [18], copolymerization [19] and interpenetrating the polymer network [20], etc. Zhou et al. [21] synthesized graft copolymers of epoxy resin and polysiloxane by a condensation reaction and then added the synthesized copolymers to the silicone resin. The test results showed that the adhesion and mechanical properties of the modified silicone resin increased. However, the epoxy resin contains fewer hydroxyl functional groups, and polysiloxane has only a small amount of reactive functional groups, so the degree and efficiency of the grafting reaction are not high, and there are some free epoxy components. Yang et al. [22] prepared a transparent silane-modified epoxy resin by a controllable sol-gel method, which showed excellent thermal stability, good adhesion and relatively low volume shrinkage. Elzaabalawy et al. [23] mixed epoxy resin with amino functionalized polysiloxane by mechanical stirring to obtain a siloxane-modified epoxy polymer. The coating prepared by this polymer has excellent adhesion and mechanical properties. Sun et al. [24] synthesized polydimethylsiloxane with aminopropyl-terminated pendant groups through ring-opening polycondensation. Next, the bisphenol A (BPA) was introduced into the polysiloxane copolymers to form the epoxy-modified polysiloxane-based resin. The coating prepared with this copolymer has excellent mechanical properties and adhesion strength.

The above-mentioned studies are helpful to improve the adhesion of silicone coating on epoxy primers. Some investigations by blending methods are simple and convenient [14,15,16], but the improvement of adhesion is limited. Others can improve their adhesion with an epoxy primer through chemical grafting, polymerization and other synthesis processes [17,18,19,20,21,22,23,24]. The preparation process is, however, complex and time-consuming. For these reasons and combining the structural characteristics of silane coupling agent and epoxy resin, silicone tie-coatings modified by both BPA and DAMO were prepared with a simple preparation method in this work. Firstly, DAMO and BPA were mixed in proportion by mechanical stirring to obtain silanized epoxy resin. Secondly, the silanized epoxy resin was added to PDMS, then the curing agent tetraethyl orthosilicate and the catalyst dibutyltin dilaurate were added to prepare the tie-coating. In this study, the tie-coating with a different epoxy content was prepared, the tensile properties and structural components of the tie-coating were investigated, and the interlaminar adhesion between the tie-coating and the matching coating was investigated.

## 2. Materials and Methods

### 2.1. Materials

Epoxy primer Penguard HB was purchased from Jotun Coatings Co., Ltd. (Zhangjiakou, China). Silicone tie-paint was made by the laboratory, hydroxy-terminated polydimethylsiloxane (PDMS) was purchased from Dayi Chemical Co., Ltd. (Yantai, China), the kinematic viscosity of PDMS is 10,000 mm^2^/s. BPA epoxy resin (E51) was obtained from Nantong Xingchen Synthetic Materials Co., Ltd. (Nantong, China), the epoxy equivalent is 184–195 g/mol. *N*-(2-Aminoethyl)-3-aminopropyltrimethoxysilane (DAMO) was purchased from New Blue Sky New Materials Co., Ltd. (Wuhan, China). Tetraethyl orthosilicate (TEOS), dibutyltin dilaurate (DBTDL) and dimethylbenzene are all of the analytical grade and purchased from Kemiou Chemical Reagent Co., Ltd. (Tianjin, China). Silicone antifouling paint was made by the laboratory.

### 2.2. Preparation of the Specimen

**Figure 1:**
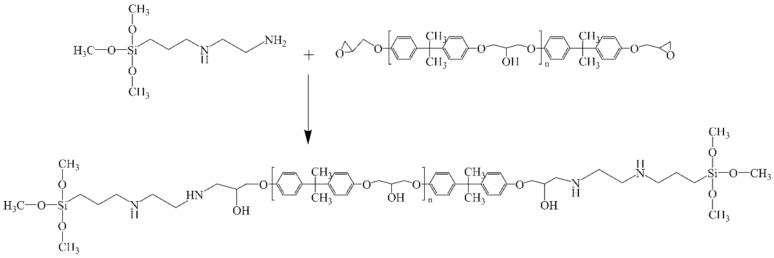
The reaction principle of DAMO and E51.

Silicone tie-paint was composed of a binder and functional agent component. The binder component was prepared by BGD 750 Versatile Sand-Milling Dispersing-agitator (Biuged Laboratory Instrument Supplies Co., Ltd., Guangzhou, China), the preparation process was as follows: DAMO and E51 were added to the BGD 750 Versatile Sand-Milling Dispersing-agitator according to the formula ratio, disperse for 10 min at a speed of 1000 rpm. The chemical reaction involved in this process is shown in Figure 1. Then, PDMS was added and the speed was increased to 3000 rpm blending for 30 min. During the period, in order that the binder component was prepared under better dispersion conditions, an appropriate amount of dimethylbenzene was added according to the state of the paint. After blending was completed, the binder was stored in a tinplate container after grinding. Before painting, the binder and functional agent components were mixed according to the formula ratio. Finally, an appropriate amount of solvent was added evenly and the coating could be prepared.

Samples were prepared according to the experimental design scheme. The formulation of the epoxy-modified silicone tie-paint is presented in Table 1, in which TEOS and DBTDL were used respectively as crosslinking curing agents and a catalyst was used to ensure that the system could be cured at room temperature. These coatings were prepared by an air spraying method to ensure that the same coatings in the samples were made with the same spraying pressure, speed, and spraying times. All the samples were flat and were placed in a high-low temperature damp heat test box at 25 °C and a humidity of 60% for curing for 7 days and were then removed for testing. The samples labeled ES-X, X represent the content (token integer) of E51 (mass%) in the formulation of the epoxy-modified silicone tie-paint.

### 2.3. Characterizations

#### 2.3.1. Microstructure and Morphology Analysis

The binder component of tie-coating was characterized by Frontier PerkinElmer infrared spectrometer (PerkinElmer Co., Ltd., Waltham, MA, USA) using the Attenuated Total Reflection (ATR) method. The scanning range is 4000–650 cm^−1^. The resolution is 2 cm^−1^. The number of scans is 32.

In order to investigate the fracture morphology between coatings, some tie-paint samples and silicone antifouling paint was prepared onto 75 mm × 25 mm × 1 mm glass slides in sequence, which had been coated with an epoxy primer. After curing, the glass slide sample was deep-frozen in liquid nitrogen for 24 h and was knocked immediately when it was taken out of the liquid nitrogen. The fracture morphology of each sample was observed with the Carl Zeiss Supra 55 Scanning Electron Microscope (SEM).

#### 2.3.2. Tensile Tests

In order to investigate the tensile properties of the tie-coating, some tie-paint samples were prepared in a Teflon mold with a dimension of 150 mm × 150 mm × 2 mm and was demolded after curing for 7 days in a high–low temperature damp heat test box. The cast film samples were cut into 45 mm × 4 mm dumbbell-shaped samples with a dumbbell-shaped knife. According to the national standard GB/T 528-2009 (ISO37-2005), samples were tested with the UTM 5105 computer-controlled electronic universal testing machine (Jinan Wance Electrical Equipment Co., Ltd., Jinan, China). The tensile speed was 50 mm/min. For each tie-coating, three reduplicate measurements were performed. An average of the elastic modulus, fracture elongation and fracture strength were calculated and analyzed.

#### 2.3.3. DSC Analyses

The structural components of the tie-coating were investigated by Differential Scanning Calorimeter; 5~10 mg of the cast film samples was put into the crucible, Nitrogen was used as protective gas, and it was cooled by liquid nitrogen. The measurement was carried out at a heating rate of 5 °C/min from −100 °C to 150 °C.

#### 2.3.4. Crosslink Density

In order to investigate the crosslink density of the tie-coating, the crosslink density of the cast film sample was investigated by the equilibrium swelling method. The weighed cast film sample was put into a test tube containing 50 mL of methylbenzene, the cap was closed tightly and it was immersed in a constant temperature water bath at 25 °C. The cast film sample was taken out from methylbenzene every 3 h, the solvent on the surface was quickly absorbed with filter paper and immediately placed into the weighing bottle. The sample was weighed with a precision electronic balance and then the cast film sample was continuously swelled in the solvent until the difference between the two adjacent weighing results did not exceed 0.1 mg. The cast film sample was investigated up to the swelling equilibrium state. The crosslink density of the cast film sample can be indicated by the relative molecular mass (Mc) between the adjacent crosslinking points of the polymer. The relative molecular mass between the adjacent cross-linking points of the sample was calculated according to the Flory-Rhener method. In order to ensure the accuracy of the data, each sample was tested 6 times, and the average value was taken to represent the Mc of the sample.

#### 2.3.5. Interlaminar Shear Strength and Adhesion Test

**Figure 2:**
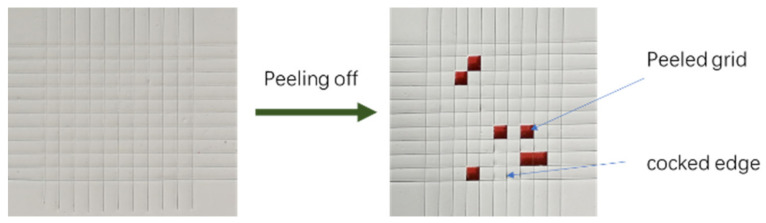
Schematic of the interfacial strength evaluation.

Some tie-paint samples were spread onto 150 mm × 70 mm × 1 mm steel plate which had been coated with an epoxy primer. A cutting tool was used to cross-cut on the samples surface with the assistance of BGD 503 Cross Cutting Rule (Biuged Laboratory Instruments Co., Ltd., Guangzhou, China). The cutting interval was 1.5 mm, the distance between the grid area and the edge of the steel plate was not less than 5 mm, cut into a grid of 100, and then the PET silicone tape was glued to the grid area and peeled off after 5 min at room temperature. It was observed and the level damage to each sample was measured (Figure 2). The adhesion between tie-coating and epoxy primer was characterized according to the anti-peeling rate (APR). The APR of the sample was obtained according to the following formula: $A\mathrm{PR}\;=\;\frac{100\;-\;\mathrm{number}\;\mathrm{of}\;\mathrm{peeled}\;\mathrm{grid}\;-\;0.25\;\times \;\mathrm{number}\;\mathrm{of}\;\mathrm{cocked}\;\mathrm{edge}}{100}\;\times \;100\%.$

**Figure 3:**
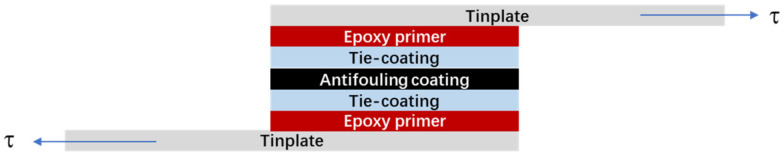
Schematic of the shear test.

The shear strength of the silicone antifouling coating/tie-coating/epoxy primer composite coatings was tested by the UTM5105 computer-controlled electronic universal testing machine (Jinan Wance Electrical Equipment Co., Ltd., Jinan, China) (Figure 3). The tensile speed was 20 mm/min. The sample preparation process was as follows: the two tin-plates with dimensions of 120 mm × 25 mm × 0.28 mm were overlapping and placed in the same direction, and the overlapping area was 30 mm × 25 mm. Epoxy primer was coated separately to the inner surface of the overlapping area of the two tin-plates. The prepared tie-paint was applied between the two epoxy primers after the epoxy primer had dried and then the silicone antifouling paint was applied to the surface of the tie-coating after the tie-coating drying, and the two parts were tied through the silicone antifouling coating.

## 3. Results and Discussion

### 3.1. The Chemical Structure of the Silicone Tie-Coatings

#### 3.1.1. ATR-FTIR

**Figure 4:**
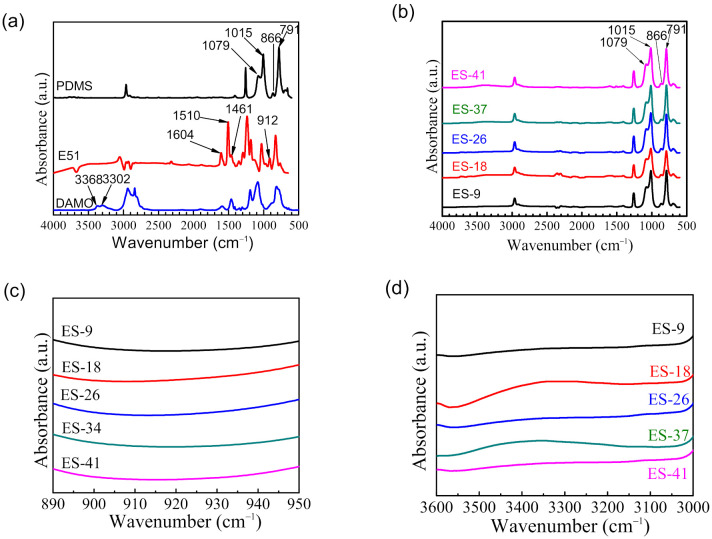
ATR-FTIR spectra of (**a**) main components in the tie-coating; (**b**) binder of tie-coating; (**c**) between 890 cm^−1^ and 950 cm^−1^ of the binder component; and (**d**) between 3000 cm^−1^ and 3600 cm^−1^ of the binder component.

The infrared spectra of the three main components in the tie-coating were shown in Figure 4a. The 3302 cm^−1^ and 3368 cm^−1^ in DAMO are symmetric and antisymmetric stretching vibrations of -NH_2_. The characteristic peak at 912 cm^−1^ in E51 is the symmetric stretching vibration absorption peak of the epoxy group. The epoxy group has a strong reactivity and can react with amino functional groups. The characteristic peaks at 1461 cm^−1^, 1510 cm^−1^ and 1604 cm^−1^ are the bending vibration absorption peaks of the benzene ring (C=C). The characteristic peaks at 1015 cm^−1^ in PDMS are attributed to the symmetrical stretching absorption peaks of Si-O-Si; the characteristic peak of 1079 cm^−1^ is the antisymmetric vibration absorption peak of Si-O-Si. The characteristic peaks at 791 cm^−1^ and 866 cm^−1^ are attributed to the characteristic peaks of the stretching vibration of the Si-Me_2_ bond.

An infrared test was performed on the binder component of the tie-coating at different ratios (Figure 4b). The infrared spectrum of the binder has similar characteristic peaks due to the component of the binder of each tie-coating being the same. It was found that the obvious characteristic peaks originally located at 1461 cm^−1^, 1510 cm^−1^ and 1604 cm^−1^ in E51 disappeared. Figure 4c,d shows the location of the infrared spectrum of the binder component and no obvious characteristic peaks in the epoxy group and amino group were found. It shows that the epoxy group of E51 had reacted with the amino group in DAMO, and it is prepared for the graft reaction with silicone resin.

#### 3.1.2. DSC

**Figure 5:**
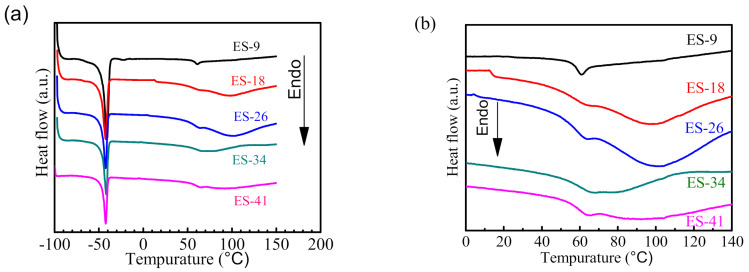
DSC curves of modified silicone tie-coatings. (**a**) measured, (**b**) part of measured.

Figure 5a shows the DSC results of the epoxy-modified silicone tie-coating. There is a step around −100 °C. Related studies have shown that the glass transition temperature of PDMS is around −123 °C [25], so it is speculated that this temperature is the glass transition temperature of the PDMS part of the tie-coating. There is an obvious melting peak around −42 °C; this is attributed to most of the film-forming substances of the tie-coating being PDMS, and the melting point of PDMS is around −45 °C, so it is inferred that this temperature is the melting point of the PDMS part of the tie-coating. This indicates that microphase separation has occurred inside the coating. The curve has a certain difference between 0 °C and 100 °C as the temperature increases (Figure 5b), which illustrates that the state of molecular motion inside the coating has changed. The epoxy resin and siloxane segments in the coating are entangled with each other, and the uniformity of the entangled network structure of the two in each coating is different. The change characteristics of this temperature range are similar to the thermal performance of epoxy resin. Kumar [26] found that the siloxane structure (-Si-O-Si-) has the thermal stability of inorganic substances. The thermal stability of epoxy resin was changed so that the thermal effect of epoxy resin was reduced by mixing with PDMS. In addition, the crosslinking density of the tie-coating increases due to the introduction of epoxy resin (Table 2), and the thermal resistance of the coating in this temperature range is different.

### 3.2. The Tensile Properties of the Silicone Tie-Coatings

**Figure 6:**
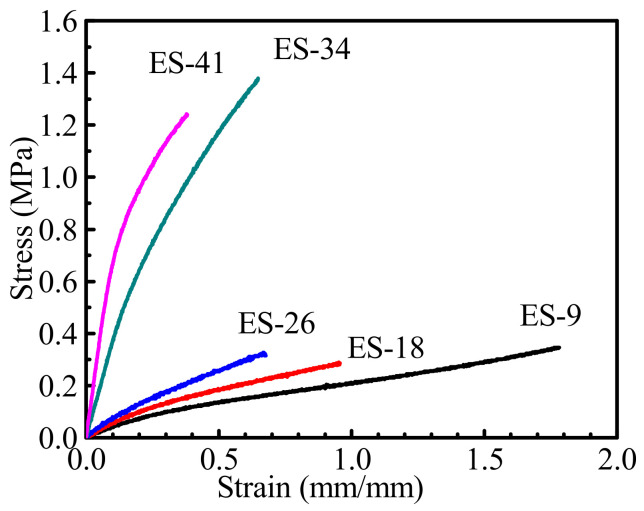
Tensile curves of modified silicone tie-coatings.

The tensile curves of the tie-coatings were shown in Figure 6 and the tensile properties of the tie-coatings were shown in the Table 3. With the increase of the epoxy resin content in tie-coating, the elastic modulus increases, the fracture elongation decreases, and the overall trend of fracture strength increases. For these reasons, on the one hand, the crosslink density of the tie-coating increased with the increasing epoxy resin content, while the Mc value decreased violently. On the other hand, there is microphase separation inside the coating, and epoxy resin is involved in the cross-linking and curing of the tie-coating. Since the rigidity of the epoxy resin is stronger than that of silicone resin, this will cause the elastic modulus of the tie-coating to be higher than that of pure silicone coating. Therefore, the higher the epoxy resin content in the tie-coating, the greater the elastic modulus of the tie-coating and the lower the elongation at the break.

### 3.3. Interlaminar Adhesion and Shear Strength

**Figure 7:**
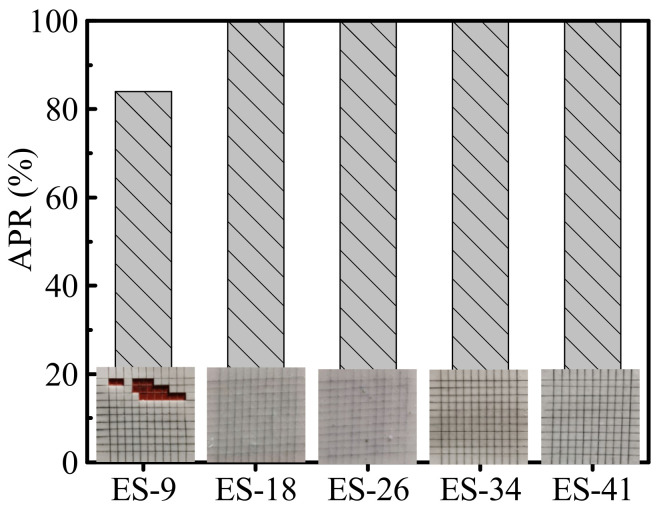
Anti-peeling rate of epoxy-modified silicone tie-coating on epoxy primer.

The adhesion of the tie-coating on the epoxy primer was shown in Figure 7. It can be seen that the tie-coating ES-9 has a regional peeling when used in conjunction with the epoxy primer. The APR of the tie-coating ES-18, ES-26, ES-34 and ES-41 on the epoxy primer is 100% and good interlaminar adhesion between the tie-coating and the epoxy primer was achieved. This phenomenon can be explained as follows: on the one hand, the epoxy structure in the tie-coating is used to tie with the epoxy primer, but the epoxy resin content in the tie-coating ES-9 is low, which means the number of epoxy structures is low in the cross-linked structure. On the other hand, the epoxy resin pre-reaction product and PDMS undergo a cross-linking reaction under the action of the curing agent TEOS and the catalyst DBTDL. There is a competitive reaction in this reaction, which is the condensation reaction between PDMS, the condensation reaction between the epoxy resin pre-reaction product and PDMS and the condensation reaction between the epoxy resin pre-reaction product. If there are no or few epoxy resin pre-reaction products to participate in the cross-linking reaction in a certain area at the bottom of the tie-coating, the interlaminar adhesion between the tie-coating and the epoxy primer is poor in this area.

**Figure 8:**
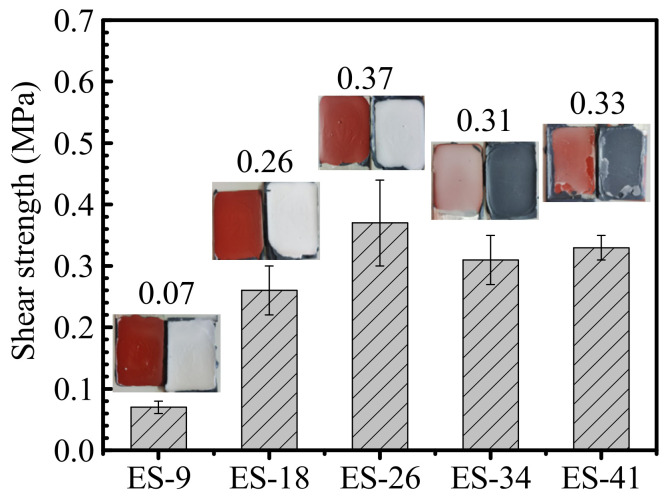
Shear strength of epoxy-modified silicone tie-coating and the matching coating system.

In the application of silicone antifouling coating, the attached organisms are removed when ships reach a certain speed. During this process, the coating was subjected to a large shear force. For this type of tie-coating, the introduction of epoxy resin will not only affect the interlaminar adhesion with the epoxy primer, but also affect the interface bonding with the silicone antifouling coating. However, the cross-cut method is not suitable for an interlaminar adhesion test between the antifouling coating and the tie-coating. In order to further investigate the connection performance of the tie-coating, a shear experiment was designed to test the shear strength of the matching coating system (epoxy primer/tie-coating/antifouling coating). This test can reflect the interlaminar adhesion between the coatings. The test result are shown in Figure 8.

It can be found that with the increase of E51 content in the tie-coating from 9% to 26%, the shear strength of the matching coating system increases from 0.07 Mpa to 0.37 Mpa. The latter is the greatest value of shear strength at breaking. Increasing the E51 content to 33.9% and 41%, the shear strength was in turn 0.31 Mpa and 0.33 Mpa. These values are higher than the maximum shear strength (0.29 Mpa) reported in the previous investigation [13]. This shows that the silicone tie-coating modified jointly by both an epoxy and a silane coupling agent has a higher interlayer bonding strength than that of the silicone tie-coating, which is modified only by the silane coupling agent. Considering the standard deviation of the measurement results, the decrease in the shear strength is not obvious. The failure position of the samples was obviously different. The failure position of ES-9, ES-18 and ES-26 are located at the interface of the epoxy primer/tie-coating. For ES-34 and ES-41, the failure position is transferred to the interface of the tie-coating/antifouling coating, which indicates that the interface bonding strength between the epoxy primer and tie-coating is higher than that between the tie-coating and antifouling coating. This is mainly due to more epoxy resin being introduced into the tie-coating, which improves the interface bonding strength between the tie-coating and the epoxy primer. Adding too much epoxy resin will, however, weaken the bonding strength between the tie-coating and silicone antifouling coating.

### 3.4. Effect of the Epoxy Resin Content on Fracture Morphology of the Silicone Antifouling Coating/Tie-Coating/Epoxy Primer Composite Coatings

**Figure 9:**
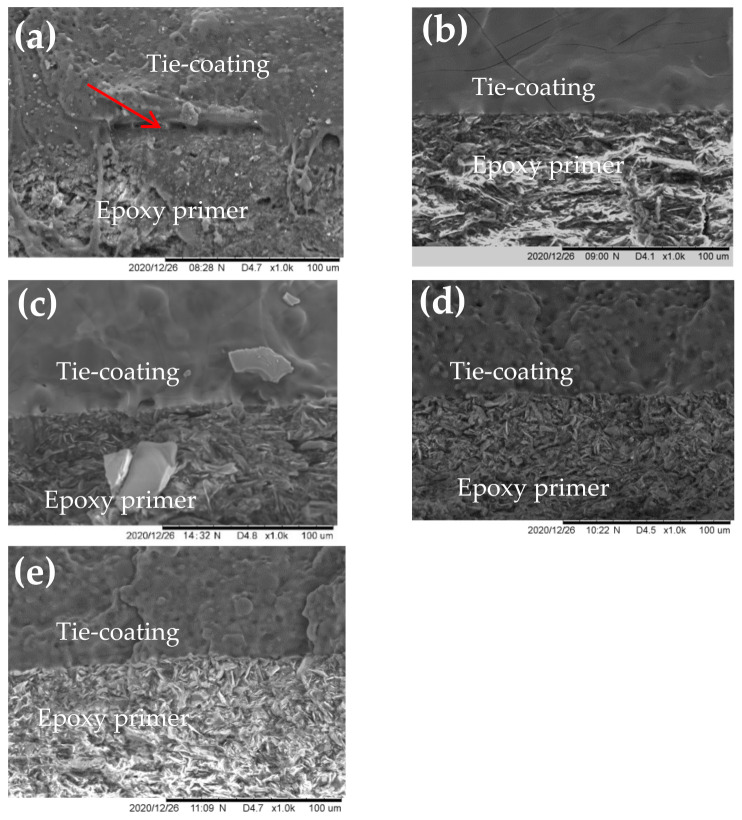
SEM images of fracture (Epoxy primer /Tie-coating) (**a**) ES-9, (**b**) ES-18, (**c**) ES-26, (**d**) ES-34, (**e**) ES-41.

The fracture morphology can intuitively reflect the connection status between the coatings. Observing the details of the fracture through the SEM can evaluate the interlaminar adhesion between matching coating. The SEM images of the fracture morphology of the epoxy primer and the tie-coating were shown in Figure 9. It can be observed that there are defects in some areas between the epoxy primer and the tie-coating in ES-9 (Figure 9a), which will result in a poor interlaminar adhesion between two coatings. The connection between the epoxy primer and the tie-coating in ES-18, ES-26, ES-34 and ES-41 is relatively good (Figure 9b–e), which means the interlaminar adhesion between the two coatings is better. This result is consistent with the test results found via the cross-cut method.

**Figure 10:**
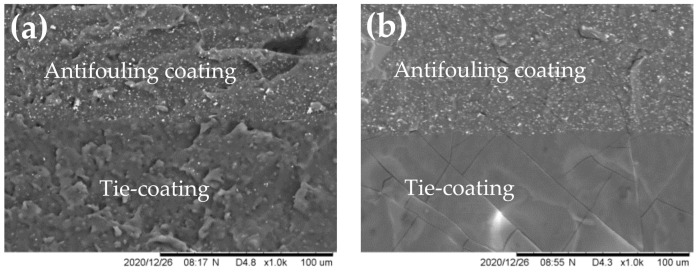

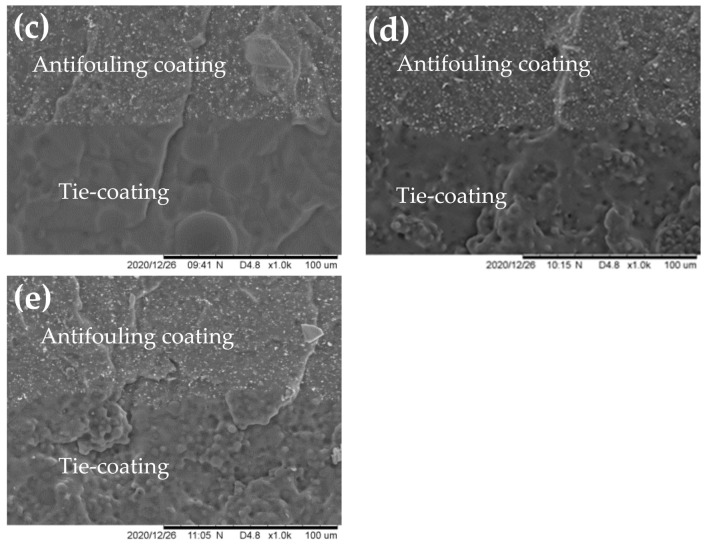
SEM images of fracture (Antifouling coating /Tie-coating). (**a**) ES-9, (**b**) ES-18, (**c**) ES-26, (**d**) ES-34, (**e**) ES-41.

The SEM images of the fracture morphology of the antifouling coating and the tie-coating are shown in Figure 10. It can be seen intuitively that the interface connection between the tie-coating and the antifouling coating is better. However, combined with the shear strength test, it can be seen that the failure position of ES-34 and ES-41 is located at the interface between the tie-coating and the antifouling coating. This was attributed to the fact that the interface bonding strength between the epoxy primer and the tie-coating in ES-34 and ES-41 is greater than the interface bonding strength between the tie-coating and the antifouling coating. The failure position is easy to find where the interface bonding strength is relatively weak when damaged by external force. As a tie-coating, the analysis of its performance should start from the whole matching coating system.

### 3.5. Tie Mechanism of the Silicone Tie-Coating

**Figure 11:**
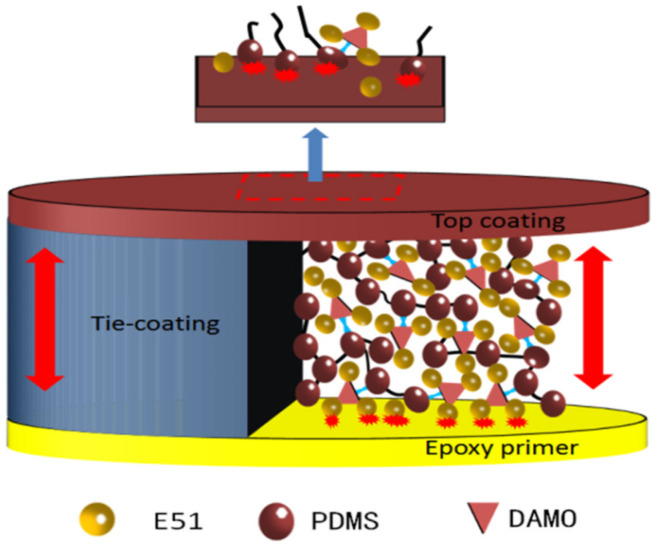
Schematic diagram of tie mechanism of the modified silicone tie-coating.

**Figure 12:**
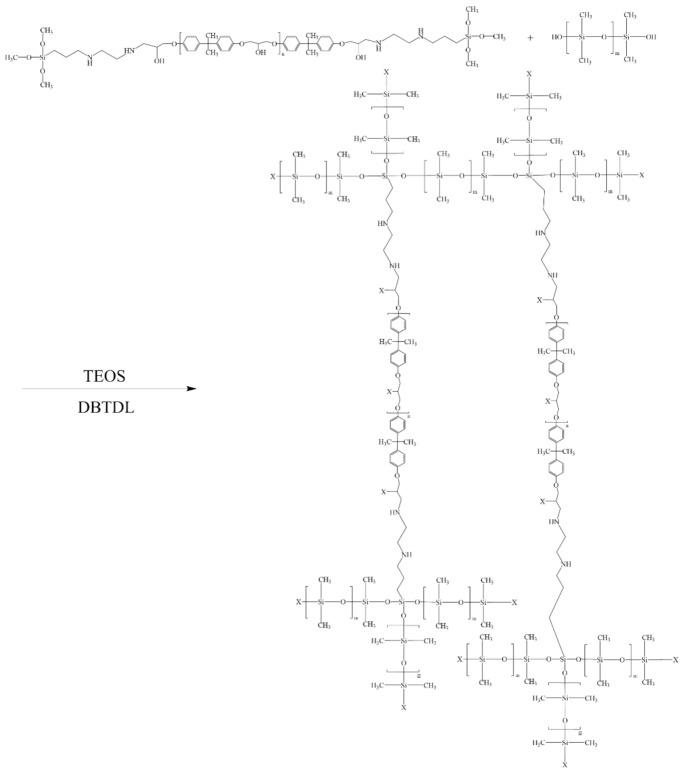
The cross-linking reaction principle of the modified silicone tie-coating.

The coatings of the two connected parts are silicone coating and an epoxy primer, respectively. The interlaminar adhesion between the two coatings is poor due to the great difference between the properties of the two coatings. The tie-coating was designed to have the characteristics of being tied with two coatings at the same time. According to the principle of a “similar compatibility”, the tie-coating was connected with the two coatings through similar parts with the two coatings respectively. The tie mechanism of the epoxy-modified silicone tie-coating is shown in Figure 11. Due to the poor compatibility between epoxy resin and silicone resin, the compatibility of two resins was improved by using the groups in the DAMO molecular structure which can react with the two resins. Firstly, epoxy resin E51 was pretreated with the silane coupling agent DAMO to prepare for the introduction of the epoxy resin structure into the siloxane chain segment. Finally, epoxy-modified silicone tie-coating was obtained by a cross-linking reaction under the action of a curing agent and a catalyst. There is a competitive reaction at this stage, and the product is not unique. A possible reaction mode of the two substances is shown in Figure 12. Because many structures of the two substances can react with each other, there is uncertainty. Therefore, X was used to represent the uncertain chemical structure. The hydroxyl group of PDMS was grafted with the alkoxy group of the pretreatment product E51. The tie-coating has the characteristics of an epoxy coating and a silicone coating at the same time because the cross-linking structure of the tie-coating has the structure of an epoxy resin and a silicone resin. At the bottom of the tie-coating, the epoxy structure as part of the tie-coating was combined with the epoxy primer to realize the connection between the tie-coating and the epoxy primer. At the top of the tie-coating, the Si-O chain structure of the tie-coating was combined with the silicone antifouling coating to realize the connection between two coatings, so as to realize the tie between the silicone antifouling coating and the epoxy primer.

## 4. Conclusions

The silicone tie-coating modified jointly by an epoxy and a silane coupling agent was successfully prepared. Its crosslinking density increased with the increase of the epoxy content. The elastic modulus and fracture strength increased, but the elongation at the fracture decreased.

With the increase in epoxy content, the peel resistance of the tie-coating on the epoxy primer increased, as did the shear strength of the matching coating system. When the E51 content was up to 26.3%, the anti-peeling rate of the tie-coating on the epoxy primer reached 100%, and the shear strength of the matching coating system reached a maximum 0.37 MPa. It has a higher interlayer bonding strength than that of the silicone tie-coating, which is modified only by silane coupling agent.

The fracture morphology revealed that the failure position of the matching coating system changes with the epoxy content. When it was lower than 26.3%, the fracture occurred at the interface between the tie-coating and the epoxy primer due to weak interfacial bonding. When it was higher than 26.3%, the fracture occurred at the interface between the tie-coating and the silicone antifouling coating due to weak interfacial bonding.

## Figures and Tables

**Table 1:** The formulation of the epoxy-modified silicone tie-paint (mass%).

Material	PDMS	E51	DAMO	TEOS	DBTDL
ES-9	84.6	9.4	3.9	1.7	0.4
ES-18	72.5	18.1	7.5	1.5	0.4
ES-26	61.3	26.3	10.9	1.2	0.3
ES-34	50.8	33.9	14.1	1.0	0.3
ES-41	41.0	41.0	17.0	0.8	0.2

**Table 2:** Crosslink density of modified silicone tie-coatings.

Coating	ES-9	ES-18	ES-26	ES-34	ES-41
Mc	9790.56 ± 65.43	4966.69 ± 233.40	2593.19 ± 158.32	1746.49 ± 23.09	1069.32 ± 14.13

**Table 3:** Tensile properties of modified silicone tie-coatings.

Sample	Elastic Modulus (MPa)	Fracture Elongation (%)	Fracture Strength (Mpa)
ES-9	0.32 ± 0.09	178.24 ± 8.54	0.35 ± 0.04
ES-18	0.44 ± 0.07	95.62 ± 4.58	0.29 ± 0.01
ES-26	0.75 ± 0.11	76.6 ± 9.97	0.34 ± 0.02
ES-34	3.46 ± 0.45	64.86 ± 8.57	1.38 ± 0.14
ES-41	7.44 ± 0.39	38.74 ± 5.61	1.24 ± 0.29

## Data Availability

Date is contained within the article.
